# A comparison in association and linkage genome-wide scans for alcoholism susceptibility genes using single-nucleotide polymorphisms

**DOI:** 10.1186/1471-2156-6-S1-S89

**Published:** 2005-12-30

**Authors:** Yen-Feng Chiu, Su-Yun Liu, Ya-Yu Tsai

**Affiliations:** 1 Division of Biostatistics and Bioinformatics, National Health Research Institutes, Miaoli, Taiwan, ROC; 2 Center for Inherited Disease Research, Institute of Genetic Medicine, Johns Hopkins University, Baltimore, Maryland, USA

## Abstract

We conducted genome-wide linkage scans using both microsatellite and single-nucleotide polymorphism (SNP) markers. Regions showing the strongest evidence of linkage to alcoholism susceptibility genes were identified. Haplotype analyses using a sliding-window approach for SNPs in these regions were performed. In addition, we performed a genome-wide association scan using SNP data. SNPs in these regions with evidence of association (*P *≦ 0.0001) were identified. We found that the general patterns for nonparametric linkage (NPL) scores from SNP and microsatellite genome scans are fairly consistent; however, the peaks of the NPL scores are mostly higher in the SNP-based scan than those using microsatellite markers, which might be located at different regions. Furthermore, SNPs identified from linkage screens were not so strongly associated with alcoholism (the most significant SNP had a *p*-value of 0.030) as those identified from association genomic screening (the most significant SNP had a *p*-value of 2.0 × 10^-8^).

## Background

Genome-wide linkage scans are typically conducted to narrow down regions prior to association fine mapping. However, Risch and Merikangas [1] claim that linkage analysis has limited power to detect genes of modest effect, and that an association approach utilizing candidate genes has far greater power, even if one needs to examine every gene in the genome. The availability of large-scale, high-throughput genotyping has made the direct genome-wide SNP-based association studies plausible. Recently, John et al. [2] compared the utility of SNPs for linkage analysis with microsatellites. They demonstrated that dense SNP data revealed linkage signals that were not detected in a low-resolution microsatellite scan. They found that the variation in information content was the main factor contributing to observed differences in the two scans based on single-nucleotide polymorphisms (SNPs) and microsatellites, and that the presence of linkage disequilibrium (LD) between a proportion of markers did not significantly affect the analysis. However, Schaid et al. [3] showed that the presence of LD among SNPs can lead to inflated LOD scores when using current genetic-linkage software under the assumption of linkage equilibrium. Similarly, they also identified more linkage peaks with narrower widths by SNPs than microsatellite markers after excluding SNPs with high LD. Despite a few recent attempts to use SNPs in genome-wide scans, a comparison of association versus linkage analyses remains limited. Therefore, the objectives of the present study were to examine the utility of SNPs in linkage analysis when compare with that of the microsatellites markers, and to investigate the value of SNP markers in linkage and association analyses.

## Methods

### Materials

A total of 143 pedigrees (or 364 nuclear families) comprising 1,614 subjects (643 individuals with alcoholism) were analyzed. There were 328 microsatellite markers and 11,120 Affymetrix SNP markers available for analysis. To test for Hardy-Weinberg equilibrium (HWE), one subject from each pedigree was randomly sampled and a chi-square goodness-of-fit test was performed using PROC ALLELE procedure in SAS/GENETICS package. Four hundred and thirty-one SNPs and 69 microsatellites were excluded as a result of departure from HWE. To avoid potential bias caused by rare alleles, 192 SNPs with minor allele frequencies less than 0.02 were further excluded. In addition, to reduce the impact of LD on our linkage results, we computed the pairwise LD measure |D'| sequentially using FBAT computing package [4]. For any two consecutive SNPs with |D'| >0.7, only the one with higher information content (heterozygosity) was included in the analyses (3,169 additional SNPs were then excluded). As a result, only 7,328 out of 11,120 SNPs were included in linkage analysis. For the association scan, 10,187 SNPs were used in the analysis, after excluding 431 SNPs for departure from HWE, 192 SNPs with minor allele frequencies less than 0.02, and 310 SNPs on chromosome X. The phenotype used was alcoholism defined by DSM-III-R alcohol dependence and Feighner's phenotype "Alc Definite" [5].

### Linkage and association analyses

Genome-wide microsatelite or SNP linkage screens were conducted using GENEHUNTER 2.1 [6]; linkage evidence was assessed on the basis of NPL scores. Due to the limitation of maximum numbers of markers in GENEHUNTER, linkage analyses were performed for every 50 SNPs. The whole-genome association scan and multi-SNP haplotype analysis were performed using family-based association tests implemented in the FBAT computing package [4], which uses nuclear families (missing parents are allowed) to test the composite null hypothesis of no association and no linkage. A region with NPL scores greater than 3.0 was identified from the genome-wide linkage scan for haplotype analysis, aiming to test the null hypothesis of no association in the presence of linkage. A sliding-window approach [7] was employed when conducting haplotype analysis on the SNPs identified from the genome-wide linkage scan.

## Results

### Linkage analysis using SNPs

The average information content from 7,328 SNPs after excluding 3,169 SNPs was almost identical to the original 11,120 SNP markers. The peak NPL scores on individual chromosomes dropped slightly on most chromosomal regions compared to those using all the markers. For example, the peak NPL scores dropped from 3.81 to 3.72 on chromosome 2, from 2.86 to 2.59 on chromosome 4, from 3.76 to 3.08 on chromosome 10, and from 2.94 to 2.44 on chromosomes 11, respectively (Table 1). There were exceptions: the peak NPL scores rose from 1.88 to 2.13 on chromosome 3, and from 1.46 to 1.74 on chromosome 20. Most of the peaks remained located at the same regions, except for the peaks on chromosomes 1 and 6. Because the exclusion of markers did not reduce much of information content in the markers, the reduction of NPL scores could possibly be due to the violation of HWE and LD assumptions from the excluded SNPs [3].

### Comparisons of SNP and microsatellite markers

**Figure 1:**
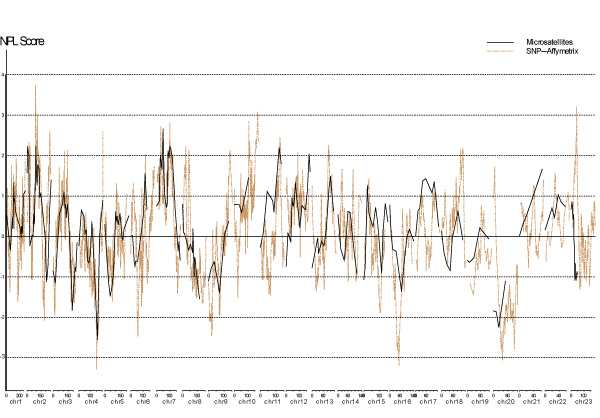
**Genome-wide scans using microsatellite and SNP markers. **NPL scores for microsatellite markers (solid line) and SNP markers (dotted line).

The overall patterns of NPL scores curves derived from microsatellites and SNP markers were fairly consistent (Figure 1). The regions identified by both types of markers (i.e., when NPL score peaks for microsatellite fell within 1-LOD support intervals constructed by SNPs) were on chromosomes 2, 6, 7, 9, 11, 13, and 15 (Table 2). Among these regions, only the signals (defined by peak NPL scores of at least 1.5) on chromosomes 2, 6, 7, and 11 were picked up by scans of both types of markers. The peaks appearing in the SNPs scan were mostly higher than those in microsatellites. For example, the corresponding peak NPL scores on chromosomes 2, 6, 7, and 11 were 2.24, 1.56, 2.22, and 2.20 for microsatellites and were 3.72, 2.03, 2.81, and 2.44 for SNPs (Table 2). Other linkage regions identified by SNP markers were mostly not found by microsatellites. This could be due to the fact that the overall average information content for SNPs is higher than that for microsatellite by 17% (0.91 versus 0.74; Table 2). Nevertheless, on chromosomes 21, where the information content remained higher for SNPs, the peak NPL scores was lower when compared to that for microsatellites. It is worth noting that the 1-LOD support intervals constructed by SNPs were narrower than those constructed by microsatellites.

### Association analysis

The 325^th^–344^th ^SNPs (tsc1155229...tsc0540301) on chromosome 2 and the 591^st^–600^th ^SNPs (tsc0549932...tsc0517919) on chromosome 10 with NPL scores greater than 3.0 (*p *< 0.0017) were selected for haplotype analysis at sliding-window sizes from 1 to 6 (results not shown). The haplotypes with an overall significance level less than a nominal level of 0.05 were constructed by the SNP of tsc1278942 (*p *= 0.024), and the interval of five SNPs (tsc1155229, tsc0781059, tsc0050143, tsc0159931, and tsc1278942) (*p *= 0.04) on chromosome 2. None of the haplotypes on chromosome 10 with an overall significance level less than 0.05 were observed. The most significant single haplotype was found to be "1 1 1 1 2 1" constructed by six SNPs (tsc0273475, tsc0336150, tsc0888957, tsc1346599, tsc1346603, tsc0574295) on chromosome 2, with a *p*-value of 0.0044. On the contrary, 15 markers across the genome have significance levels less than a nominal level of 0.0001 when testing for the null hypothesis of no association and no linkage (Table 3). Among them, the significance levels for tsc0515272 on chromosome 3, tsc0029429 on chromosome 9 and tsc1750530 on chromosome 16 were 3.8 × 10^-7^, 2.0 × 10^-8^, and 4.5 × 10^-7^, respectively, which were smaller than 4.91 × 10^-6 ^(= 0.05/10,187), the significance level of 0.05 with a conservative Bonferroni correction for 10,187 SNPs used in the association analysis. Nevertheless, none of these SNPs were located in the regions showing evidence of linkage. These results indicated that the genetic effects from the alcoholism causal variants might be too weak to be identified through linkage analysis, yet can be detected by genomic association studies.

## Conclusion

Our analyses illustrated that the typical gene-mapping procedure to identify target regions through a genome-wide linkage scan using markers at a density of 1 marker/~10 cM prior to a fine-scale mapping on the targeted regions, could possibly fail to identify disease loci due to either limited major gene effects, misplacement of markers, or insufficient information content of microsatellite markers. The initial genome-wide scan turns out to be extremely critical to select regions harboring disease genes for further fine-mapping analysis in the typical process. The availability of SNP markers provides substantially greater information content than the microsatellites, thus linkage signals missed by microsatellites could be picked up by SNPs. However, the presence of LD among the SNPs, the inability to detect Mendelian errors, and the inability to accurately validate genetic maps have complicated linkage studies using SNPs. Association studies, on the other hand, have greater power to detect genes of modest effect [1] than linkage analysis. The results from association studies, however, would need to be interpreted with caution, since numerous tests would need to be carried out in a genome-wide association scan, which would increase the false-positive rate, and a correction to significance levels for multiple tests is necessary. Additionally, in our haplotype analysis, the association between a single-SNP haplotype and alcoholism could vary substantially by the window sizes of the multi-SNP haplotypes; and the linkage signals identified in this study might not be strong enough to further identify the causative haplotypes.

## Abbreviations

HWE: Hardy-Weinberg equilibrium

LD: Linkage disequilibrium

NPL: Nonparametric linkage

SNP: Single-nucleotide polymorphism

## Authors' contributions

YFC made contributions to the study design, statistical analysis, interpretation, and draft of the manuscript. SYL participated in the design of the study and performed the data analysis. YYT conceived of the study and helped to draft the manuscript. All authors read and approved the final manuscript.

## Figures and Tables

**Figure 2:**
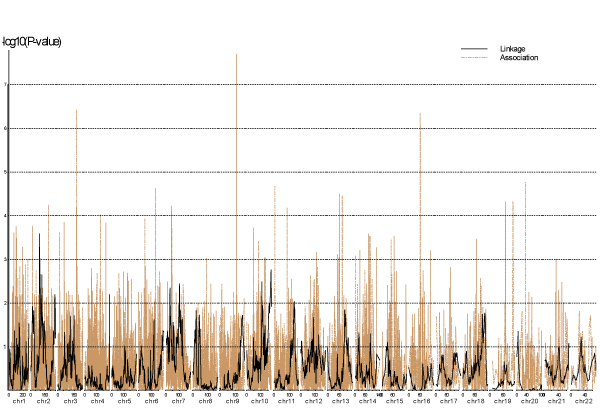
**Genome-wide linkage and association scans. **Negative of logarithm (base 10) for *p*-values from linkage (solid line) and association (dotted line) analyses using SNP markers.

**Table 1:** Comparisons of information content and NPL scores between 11,120 SNPs (All) and 7,328 SNPs (Subset) in genome-wide scans, regions with maximum NPL scores ≧ 1.5 were reported.

	Average information content		Peak NPL score		Position (cM)	
			
Chr	All	Subset	All	Subset	All	Subset
1	0.91	0.91	**1.81^a^**	1.75	77.15	77.15
			1.79	1.54	107.16	107.16
			1.56		126.23	
			2.04	1.95	142.09	142.09
			2.13		237.83	
			1.75	1.92	274.27	274.27
2	0.93	0.92	2.72		5.74	
			2.44	2.33	18.03	18.03
			3.81		90.58	
				3.72		90.60
			2.96	2.99	112.07	112.07
				1.98		135.497
			2.31		136.45	
			2.74	2.59	244.44	244.44
3	0.93	0.91		1.70		60.44
			1.80		60.44	
			1.84		86.23	
				2.10		111.82
			1.88	2.13	143.25	144.53
4	0.92	0.92	2.86	2.59	204.47	204.47
5	0.92	0.92	1.52		109.78	
6	0.89	0.89	2.06		21.02	
			1.92	2.03	116.07	116.07
			2.03	1.74	185.42	185.42
7	0.91	0.92	1.98	1.74	13.65	13.65
				2.72		31.68
			2.86		31.84	
			2.23		54.06	
				1.96		54.08
			2.90	2.81	101.05	101.05
			1.98	2.04	123.79	123.79
			2.08		128.61	
8	0.92	0.92	2.29	2.24	21.97	21.97
			2.10		41.70	
				2.14		42.16
9	0.92	0.91	2.24	2.13	141.17	141.17
10	0.91	0.90		1.57		47.31
			1.71		48.75	
			2.74	2.85	106.69	106.69
			2.03	1.87	128.63	128.63
			3.76	3.08	171.41	171.41
11	0.91	0.92		2.44		125.15
			2.94		125.27	
12	0.92	0.91	1.58		4.76	
			1.53		51.77	
			1.88		71.83	
			2.06	2.08	86.43	86.43
			1.71		107.48	
				1.64		122.16
			1.60		122.20	
			1.85		169.44	
13	0.83	0.92	1.87		0.0000	
			2.56	2.26	86.87	86.87
14	0.92	0.91	1.81	1.66	23.73	23.73
			1.71	1.66	88.50	88.50
15	0.91	0.91	1.62		29.81	
16	0.90	0.89				
17	0.90	0.89				
18	0.92	0.91		2.19		106.82
			2.35		108.45	
19	0.88	0.87				
20	0.90	0.90		1.74		5.81
21	0.92	0.91				
22	0.87	0.87	1.61	1.57	28.51	28.50
23	0.91	0.91	3.21	3.21	42.78	42.78
Overall average SD	0.91 (1.18)	0.91 (0.094)				

^a^Gray text indicates local maximums in the region, while black text indicates the global maximum.

**Table 2:** Comparisons of regions with peak NPL scores ≧ 1.5 by chromosomes using microsatellite and SNP markers.

	Microsatellite				SNP			
		
Chr	Average information content	Peak NPL score	Position (cM)	1-LOD SI	Average information content	Peak NPL score	Position (cM)	1-LOD SI
1	0.78				0.91	**1.75^a^**	77.15	
						1.54	107.16	
						1.95	142.09	[139.92, 152.58]
						1.92	274.27	
2	0.78	2.23	4.9	[89.05, 133.01]	0.92	2.33	18.03	
		2.24	94.2			3.72	90.60	[88.14, 95.20]
						2.99	112.07	
								
						1.98	135.49	
						2.59	244.44	
3	0.71				0.91	1.70	60.44	
						2.10	111.82	
						2.13	144.53	[137.26, 144.57]
4	0.71				0.92	2.59	204.47	[204.32, 204.47]
5	0.74				0.92			
6	0.67	1.56	112.9	[99.83, 127.70]	0.89	2.03	116.07	[110.71, 117.21]
						1.74	185.42	
7	0.84	2.66	49.3	[40.96, 53.37]	0.92	1.74	13.65	
		2.22	100.0	[82.55, 133.43]		2.72	31.68	
						1.96	54.08	
								
						2.81	101.05	[93.83, 107.52]
						2.04	123.79	
8	0.76				0.92	2.24	21.97	[9.69, 24.13]
						2.14	42.16	
9	0.68				0.91	2.13	141.17	[128.25, 151.24]
10	0.64				0.90	1.57	47.31	
						2.85	106.69	
						1.87	128.63	
						3.08	171.41	[153.97, 175.87]
11	0.67	2.20	119.8	[99.24, 133.20]	0.92	2.44	125.15	[118.83, 130.89]
12	0.77	2.04	169.8	[154.18, 177.30]	0.91	2.08	86.43	[80.88, 88.63]
						1.64	122.16	
13	0.74	1.49	97.3	[80.03, 113.50]	0.92	2.26	86.87	[83.80, 97.69]
14	0.72				0.91	1.66	23.73	[17.41, 25.95]
						1.66	88.50	
15	0.75				0.91			
16	0.69				0.89			
17	0.66				0.89			
18	0.65				0.91	2.19	106.82	[92.47, 111.48]
19	0.66				0.87			
20	0.73				0.90	1.74	5.81	[0.16, 7.60]
21	0.76	1.66	62.7	[25.20, 62.70]	0.91			
22	0.62				0.87	1.57	28.51	[22.77, 34.11]
23	0.82				0.91	3.21	42.78	[36.37, 43.43]
Overall average (SD)	0.74 (0.095)				0.91 (0.094)			

^a^Gray text indicates local maximums in the region, while black text indicates the global maximum.

**Table 3:** SNPs with *p*-value ≤ 0.0001 in the family-based association tests

Chromosome	SNPs name	Allele frequency	| *Z *|	*p*-value	Position (cM)
2	tsc0052126	0.29	4.03	5.6 × 10^-5^	177.98
3	tsc0515272	0.11	5.08	3.8 × 10^-7^	164.24
3	tsc1519933	0.32	4.01	6.2 × 10^-5^	167.43
4	tsc0741686	0.047	3.90	9.5 × 10^-5^	130.23
6	tsc1175206	0.30	4.23	2.4 × 10^-5^	127.58
7	tsc0593964	0.36	4.01	6.0 × 10^-5^	42.62
9	tsc0029429	0.041	5.61	2.0 × 10^-8^	103.21
11	tsc0569292	0.22	4.26	2.1 × 10^-5^	6.78
11	tsc0919042	0.17	3.99	6.5 × 10^-5^	81.19
13	tsc0271621	0.17	4.16	3.2 × 10^-5^	60.17
13	tsc0056748	0.17	4.14	3.5 × 10^-5^	73.99
16	tsc1750530	0.13	5.05	4.5 × 10^-7^	59.83
19	tsc1189131	0.39	4.07	4.8 × 10^-5^	68.94
19	tsc0598556	0.31	4.07	4.7 × 10^-5^	102.05
20	tsc0060446	0.19	4.30	1.7 × 10^-5^	35.45
